# Prevalence of perceived stress and coping strategies among healthcare workers during the COVID-19 outbreak at Bangkok metropolitan, Thailand

**DOI:** 10.1371/journal.pone.0270924

**Published:** 2022-07-08

**Authors:** Pataraporn Yubonpunt, Jadsada Kunno, Busaba Supawattanabodee, Chavanant Sumanasrethakul, Budsaba Wiriyasirivaj

**Affiliations:** 1 Department of Public Health, Faculty of Public and Environmental Health, Huachiew Chalermprakiet University, Samut Prakan, Thailand; 2 Department of Research and Medical Innovation, Faculty of Medicine Vajira Hospital, Navamindradhiraj University, Bangkok, Thailand; 3 Department of Urban Medicine, Faculty of Medicine Vajira Hospital, Navamindradhiraj University, Bangkok, Thailand; 4 Department of Obstetrics and Gynecology, Faculty of Medicine Vajira Hospital, Navamindradhiraj University, Bangkok, Thailand; The University of Mississippi Medical Center, UNITED STATES

## Abstract

**Background:**

Healthcare workers (HCW), who are crucial workforce, have experienced stress during the COVID-19 pandemic. They have been learning to fight against and support patients as much as possible. Thus, this study aims to account for the psychological impact of the COVID-19 outbreaks on the healthcare workers of medical school hospitals in terms of their perceived stress and coping styles.

**Method:**

This cross-sectional study was conducted from June to August, 2021. 517 HCWs self-administered the online survey. Perceived Stress Scale (PSS-10) in Thai-version was used to examine the perceived stress symptoms. Brief-COPE score was used to determine the coping strategies. Independent sample t-test, one-way analysis of variance (ANOVA), and multivariable regression analysis were utilized. The level of significance was set at p-value < 0.05.

**Result:**

The prevalence of perceived stress among the HCWs was 41.97%. Coping strategies were used to deal with stress during the outbreak for problem-solving (Mean ± SD = 0.25 ± 0.60) and positive attitude (Mean ± SD = 2.85 ± 0.62). Significant difference was observed in the use of coping strategies among those who differ in marital status (F2, 514 = 7.234, p-value = 0.001), having children (t515 = -4.175, p-value < 0.001), and days off (t515 = -1.992, p-value = 0.047). Multivariable regression analysis reported who those perceived stress symptoms using social support more than those normal stress (AOR 1.54, 95% CI 1.070–2.236, p-value = 0.02). The perceived stress symptoms group used the avoidance strategy 2.03 times more than the other group (AOR 2.03, 95% CI 1.406–2.934, p-value < 0.001). Interestingly, the participants who perceived stress symptoms applied a positive attitude strategy lesser than those who experienced normal stress (57.5%) (AOR 0.42, 95% CI 0.307–0.590, p-value < 0.001).

**Conclusion:**

The impact of the COVID-19 pandemic on mental distress remains. The findings of this study suggest further study to assess the HCWs’ stress after the pandemic. HCWs should consider merging each of the coping strategies to balance work and lifestyle in pandemic situations.

## Introduction

Coronavirus or COVID-19 was declared a pandemic. COVID-19 directly affects the health and normal lifestyle of people. Healthcare workers (HCWs) are key to responding to this pandemic, as well as others. They have been doing their best to learn to fight against and support patients. Regardless of the number of patients, HCWs have exhibited similar dedication to their care. The pattern of the normal way has changed to a new normal. This is a good concept to protect and prevent people from contracting the coronavirus, although it can completely affect their emotions and lifestyle. This is also applicable to the practice of HCWs whose care process requires adherence to standard precaution. Besides, social distancing restricts their casual contact with other people including their family and friends. These make people feel isolated and lonely; this can increase stress. (CDC, 2021) [1].

The stress experienced by HCWs during COVID-19 was studied worldwide. Previous studies associated this stress to factors such as age, gender, compensation, and child [2–4]. Thus, work experience, work hours, family factors, and caring for the COVID-19 patients were mentioned as sources of stress [5, 6]. Balancing the mental health is crucial for people who face uncontrollable events, especially the stress levels that require the use of appropriate strategies. Problem-solving, avoiding, and seeking social support were stated as the coping strategies applied by the HCWs to deal with stress during a tough time of the outbreak [7, 8].

Coping refers to efforts used to prevent or reduce threats, losses, or suffering. This process can also be a protective factor because the efforts used to manage excessive stress can reduce psychological distress, which has negative consequences on physical health over short and long periods [9]. Social support has been considered an important factor that alleviates psychological stress among nurses in crises [10]. In problem solving, the individual perceives that the stressor is something on which an action can be taken for resolution. Problem-focused coping activities are utilized by individuals who perceive problems as “opportunities for benefit or gain, believe that problems are solvable” [11]. The avoidance strategy involves both the negative and positive actions used based on the situation. In unpredictable situations, avoidance coping strategies are more likely to be utilized [7]. Stress can cause biological responses such as blood pressure, changes in the sleep pattern, and cardiovascular risk. There was relation with coping strategies that protect both mental and physical health from the negative effects of stress [12]. Therefore, to be able to work in uncontrollable and risky situations, HCWs need to learn and adapt each coping strategy to manage their feelings and emotions in various situations to attain a work-life balance during the COVID-19 outbreak.

From the first to the third wave, the HCWs in Thailand have fully faced COVID-19 up till now. Bangkok is not only the center of pandemics but also the center of care. A medical school hospital functions as a healthcare setting for patients in an urban area. HCWs in this setting have more burden and workload, compared to other areas, especially the rural areas. They provide services for both COVID-19 and general patients. Their daily life stays with worse situations for a long time. Some contracted the virus, while the others were quarantined. Due to limited workforce and materials, some clinical workers were assigned to the cohort ward to treat COVID-19 patients. Their varied skills influenced their confidence and practice. Meanwhile, the support staff was assigned to help with activities involving COVID-19 management, while they went to work regularly. Although they work in different conditions, some conditions cause mental health problems. The long period of living and working under pandemic situations have caused mental health problems, of which stress is clearly deducible. Previous studies reported the prevalence of stress at different levels but mostly in a moderate to high level [13–15]. Although the mental health of HCWs was studied widely, most studies were conducted in the first period of the outbreak. Interestingly, the impact of mental health on HCWs still appears to be a concerning issue. Although HCWs protect themselves from the coronavirus with personal protective equipment and adhere to precaution guidelines, their mental health must also account for the impact of COVID-19.

Among HCWs, little is known about the prevalence of perceived stress and the strategies to cope with such stress during the outbreak in medical school hospitals, which was the center of the pandemic in Thailand during the third wave. It is imperative that we identify the coping strategies utilized during the third wave of the outbreak. This study can be a lesson for future crises. Therefore, the psychological impact of the COVID-19 outbreaks on the HCWs of medical school hospitals in terms of perceived stress and coping style is accounted as the purpose of this study. Despite the same pandemic situation, we hypothesized that different coping strategies prevail among HCWs based on various socio-demographic characteristics, work factors, and perceived stress. Besides, this study examines the coping strategies used and associated with the symptoms of perceived stress in HCWs during the pandemic.

## Methods

### Study design

This study conducted a cross-sectional survey of HCWs working at the Faculty of Medicine at the Vajira Hospital in Bangkok, Thailand. Data collection occurred between July and August, 2021. This study was approved by the ethics committees of the Faculty of Medicine at Vajira Hospital, Navamindradhiraj University, Bangkok, Thailand and Faculty of Public and Environmental Health at Huachiew Chalermprakiet University, Samutprakan, Thailand, (COA 116/2564) and (อ.1102/2564 ลว), respectively.

### Participants

HCWs aged 18 years and above agreed to participate in the study (n = 517). All participants were of Thai nationality and living in the urban community of Bangkok. The sample size was calculated using G*Power based on the estimated population of HCWs in the city.

### Data collection

The questionnaires were completed using an online survey (Google Forms). Participants were recruited on social media using a snowball technique such as Google form based on social distancing during the COVID-19 pandemic. The invitation asked for the confirmation of an informed consent from all the participants, required voluntary participation, and provided instructions for filling in the questionnaire. All participants have been performed in accordance with the Declaration of Helsinki and have been approved by an appropriate ethics committee.

### Questionnaire

The questionnaire was designed based on a previous study [12] and factors contributing to HCWs. It was adapted to the situation in Thailand by a team of experts. The questionnaire requires about 20 minutes to complete and is divided into three sections **(see S1 File for details)**. The first part involves the socio-demographic characteristics, work factors, and having contracted COVID-19, the variables of which include gender, age, education, job position, marital status, income, having children, underlying diseases, family diseases, residence, work experience, work hours, number of days off, sleep hours, work members, and the experience in itself of contracting COVID-19. The second part involves Perceived Stress Scale (PSS-10) that assesses the perceived stress of HCWs during the COVID-19 outbreak. The original questions of PSS-10 were created by Cohen et al. [16], and were widely used to measure stress in various situations. We used the Thai versions of PSS-10, which were translated by Thai researchers, with a reliability measure of 0.84 [17]. The questions were adopted by adding the COVID-19 wording. Scoring response was ranged from 0 (Never) to 4 (very often), where the higher score indicated the higher level of perceived stress in the previous month. This study defined the scoring of perceived stress symptoms to range between 21 and 40 [16, 18]. The content validity was examined by three experts and the IOC was 0.93. The present study reported a Cronbach’s alpha value of 0.751. The third part involves Brief-COPE Score to assess the coping strategies. This assessment was applied in the healthcare setting to examine the coping styles of patients and medical professional staff [15]. The previous studies about the coping style of HCWs during the COVID-19 situation reported the reliability of the Brief-COPE question to be between 0.81 and 0.85 [8, 15]. In the Thai version, the Brief-COPE assessment was translated and the same reported a reliability measure of 0.84 [19]. There were twenty-eight items with response scores ranging from 1 (I haven’t been doing this at all) to 4 (I’ve been doing this a lot). The higher the Brief-COPE score, the higher the application of coping strategies. The content validity was examined by three experts and the IOC was 0.86. The present study reported a Cronbach’s alpha value of 0.835.

### Statistical analysis

The descriptive statistics including frequency, percentages, mean, and standard deviations were analyzed to describe the characteristics, PSS-Score, and Brief-COPE score of the HCWs. Independent sample t-test and one-way analysis of variance (F-test) were used to compare the coping strategies based on differences in the socio-demographic characteristics, work factors, and perceived stress scores. Multiple linear regression analysis was run to determine the factor that can predict the need to use coping strategies in general. Durbin-Watson statistics reported no violation of multicollinearity. Multivariable logistic regression analysis was run to find an association between the domains of coping strategies and perceived stress symptoms. Statistical analysis was performed using the Statistical Package for the Social Sciences Program (SPSS), version 22. The level of statistical significance was considered at p-value < 0.05.

## Results

A total of 517 questionnaire responses were obtained, and the socio-demographic is presented in **Table 1**. The largest group of participants were female (83.4%), aged more than 35 years (59.4%), majority had an education qualification of a bachelor’s degree or higher (76.2%), the majority were single (52.8%), with reported incomes of more than 25,000 Thai Baht (50.8%), and no children in the household (58.4%). Additionally, most were medical staff (55.3%), with a reported work experience of more than 10 years (57.3%), most having worked less than 8 hours/day (65.7%), reported day off more than 8 days/month (61.3%), and slept less than 6 hours/day (61.5%).

**Table 1:** Socio-demographic and comparison of coping strategies (Brief-COPE score) among HCWs during COVID-19 (n = 517).

Socio-demographic		Comparison using of coping strategies		
Variables	n (%)	Mean ± SD	t/F	p-value
**Gender** ^a^				
Male	86(16.6)	2.54 ± 0.37	0.297	0.766
Female	431(83.4)	2.53 ± 0.36		
**Age** ^a^				
≤ 35 years	210(40.6)	2.49 ± 0.38	-0.192	0.054
> 35 years	307 (59.4)	2.56 ± 0.34		
**Education** ^a^				
Under bachelor’s	123(20.7)	2.57 ± 0.40	1.273	0.204
Bachelor’s and upper	394(76.3)	2.52 ± 0.35		
**Job Designation** ^a^				
Medical staff	286(55.3)	2.53 ± 0.35	0.152	0.879
Support staff	231(44.7)	2.53 ± 0.38		
**Marital Status** ^b^				
Single	273(52.8)	2.48 ± 0.36	7.234	0.001*
Married	208(40.2)	2.60 ± 0.35		
Separate	36(6.9)	2.56 ± 0.32		
**Income** ^a^				
≤ 25,000 baht	254(49.1)	2.53 ± 0.38	0.196	0.844
> 25,000 baht	263(50.9)	2.53 ± 0.34		
**Having Children** ^a^				
No	339(58.4)	2.48 ± 0.36	-4.17	<0.001*
Yes	178(41.6)	2.61 ± 0.35		
**Underlying Diseases** ^a^				
No	339(65.6)	2.52 ± 0.37	-0.897	0.370
Yes	178(34.4)	2.55 ± 0.34		
**Family Diseases** ^a^				
No	188(36.4)	2.51 ± 0.39	-0.905	0.366
Yes	329(63.6)	2.54 ± 0.34		
**Residence** ^a^				
Home	272(52.6)	2.54 ± 0.35	0.647	0.524
Condo-apartment	180(34.8)	2.51 ± 0.38		
Hospital	65(12.5)	2.55 ± 0.35		
**Work Experience** ^a^				
≤ 10 years	221(42.8)	2.50 ± 0.39	-1.813	0.070
> 10 years	296(57.2)	2.56 ± 0.33		
**Work Hours/day** ^a^				
≤ 8 hours	340(65.8)	2.54 ± 0.36	0.579	0.563
> 8 hours	177(34.2)	2.52 ± 0.36		
**Day off/month** ^a^				
< 8 days	200(38.7)	2.49 ± 0.39	-1.992	0.047*
≥ 8 days	317(61.3)	2.56 ± 0.33		
**Sleep Hours/day** ^a^				
≤ 6 hours	318(61.5)	2.54 ± 0.34	0.595	0.552
> 6 hours	199(38.5)	2.52 ± 0.39		
**Number of co-workers** ^a^				
≤ 3 Members	430(83.2)	2.53 ± 0.35	-0.765	0.444
> 3 Members	87(16.8)	2.56 ± 0.39		
**COVID-19 Experience** ^b^				
No risk	173(33.4)	2.54 ± 0.36	0.265	0.851
Not sure	149(28.8)	2.51 ± 0.37		
Quarantined and investigated	179(34.6)	2.53 ± 0.35		
COVID-19 Case	16(3.1)	2.57 ± 0.30		
**Prevalence of perceived stress**				
Normal stress	300(58.1)	2.51 ± 0.37	-1.680	0.094
Stress symptom	217(41.9)	2.56 ± 0.34		

^a^Independent t-test

^b^One-way analysis of variance (ANOVA)

*Statistical significant level < 0.05.

In terms of coping strategies (Brief-COPE score) during the COVID-19 characteristics, the result showed that marital status significantly affected the coping strategies, compared to single and separate (F2, 514 = 7.234, p-value = 0.001), the presence of children in the household significantly affected the coping strategies, compared to those who had no children (t515 = -4.175, p-value < 0.001), and having days off more than 8 days/month significantly affected the coping strategies, compared to those who had days off less than 8 days/month (t515 = -1.992, p-value = 0.047) in **Table 1**.

Hence, the prevalence of perceived stress among HCWs was 41.97% and it is shown in Table 1.

### Coping strategies (Brief-COPE score) among HCWs during the COVID-19 pandemic

Table 2 depicts the items score of the coping strategies of participants. From descriptive statistics analysis, 517 participants revealed the coping strategies they used to deal with stress during the COVID-19 outbreak. The majority, 41.97% (n = 217), of participants responded to the perceived stressed symptoms indicated by perceived stress scale (21–40 score). The presence of social support as a solution to stress (Mean ± SD = 2.67 ± 0.55) indicated that participants had a medium use of coping strategies. Problem-solving (Mean ± SD = 3.25 ± 0.60) indicated that participants had a high use of coping strategies. The use of avoidance strategy (Mean ± SD = 1.94 ± 0.0.52) indicated that few participants used coping strategies. Positive attitude (Mean ± SD = 2.85 ± 0.62) indicated that participants had a medium use of coping strategies.

**Table 2:** Descriptive statistics of coping strategies (Brief-COPE score) by dimensions and items.

Coping Strategies Items	Mean	SD
**Social Support**	**2.67**	**0.55**
1. I’ve been getting comfort and understanding from someone.	2.87	0.83
2. I’ve been getting help and advice from other people.	2.90	0.82
3. I’ve been saying things to let my unpleasant feelings escape.	2.85	0.82
4. I’ve been getting emotional support from others.	2.87	0.83
5. I’ve been trying to get advice or help from other people about what to do.	2.72	0.85
6. I’ve been expressing my negative feelings.	2.27	0.82
7. I’ve been praying or meditating.	2.28	1.05
8. I’ve been trying to find comfort in my religion or spiritual beliefs.	2.61	1.00
**Problem Solving**	**3.25**	**0.60**
9. I’ve been taking action to try to make the situation better.	3.24	0.72
10. I’ve been concentrating my efforts on doing something about the situation I’m in.	3.23	0.68
11. I’ve been trying to come up with a strategy about what to do.	3.30	0.70
12. I’ve been thinking hard about the steps to take.	3.23	0.71
**Avoidance**	**1.94**	**0.52**
13. I’ve been using alcohol or other drugs to help me get through it.	1.64	0.97
14. I’ve been using alcohol or other drugs to make myself feel better.	1.64	0.96
15. I’ve been criticizing myself.	1.86	0.85
16. I’ve been blaming myself for things that happened.	1.69	0.80
17. I’ve been refusing to believe that it has happened.	1.75	0.88
18. I’ve been saying to myself “this isn’t real”.	1.55	0.83
19. I’ve been doing something to think about it less, such as going to movies, watching TV, reading, daydreaming, sleeping, or shopping.	2.96	0.88
20. I’ve been giving up the attempt to cope.	1.69	0.79
21. I’ve been turning to work or other activities to take my mind off of things.	2.90	0.81
22. I’ve given up trying to deal with it.	1.74	0.83
**Positive Attitude**	**2.85**	**0.62**
23. I’ve been making jokes about it.	2.34	0.98
24. I’ve been making fun of the situation.	2.47	0.94
25. I’ve been learning to live with it.	3.15	0.74
26. I’ve been accepting the reality of the fact that it has happened.	3.26	0.74
27. I’ve been trying to see it in a different light, to make it seem more positive.	2.98	0.77
28. I’ve been looking for something good in what is happening.	2.91	0.78

Mean of Brief-COPE score (1.00–2.00) = low use of coping strategies.

Mean Brief-COPE score (2.01–3.00) = medium use of coping strategies.

Mean Brief-COPE score (3.01–4.00) = high use of coping strategies.

### Association between factor variables and overall Brief-COPE score

**Table 3** shows the association of the factor variable and overall Brief-COPE score among HCWs during the COVID-19 pandemic based on multiple linear regression models. Participants who had children in their household (vs. had no children in household, β 0.143; 95% CI 0.007, 0.201) were more associated with the overall Brief-COPE score.

**Table 3:** Socio-demographic variables associated with overall Brief-COPE score.

Socio-demographic variables	Overall Brief-COPE score	
	β (95% CI)	p-value
Age		
≤ 35 years	Ref.	
> 35 years	-0.004 (-0.104, 0.098)	0.951
Education		
Under bachelor’s	Ref.	
Bachelor’s and upper	-0.009 (-0.083, 0.067)	0.840
Have children		
No	Ref.	
Yes	0.143 (0.007, 0.201)	0.035*
Work experience		
≤ 10 years	Ref.	
> 10 years	0.005 (-0.095, 0.102)	0.948
Day off		
< 8 days	Ref.	
≥ 8 days	0.078 (-0.006, 0.121)	0.076
Marital Status		
Single	Ref.	
Married	0.057 (-0.056, 0.139)	0.400
Separate	-0.009 (-0.163, 0.137)	0.868

Data were analyzed using the multiple linear regression models. Data were presented as β coefficients and 95% confidence interval (CI).

*Statistical significant level < 0.05.

### Comparative difference of the use of coping strategies among HCWs during the COVID-19 pandemic

**Table 4** shows the comparison of the use of coping strategies by participants who perceived normal stress, as opposed to those who perceived stress symptoms. There was no difference overall. However, this finding indicated the significant difference in the domains of social support (p-value = 0.046), avoidance (p-value < 0.001), and positive attitude (p-value < 0.001) between those who perceived stress symptoms and those who perceived normal stress.

**Table 4:** Comparison of the use of coping strategies based on the difference of perceived stress symptoms.

Domain of coping strategies	Perceived Stress Scale		t	95% CI	p-value
	Perceived normal stress	Perceived stress symptoms			
	Mean ± SD	Mean ± SD			
Social support	2.63 ± 0.56	2.73 ± 0.53	-2.001	-0.093, -0.001	0.046*
Problem Solving	3.23 ± 0.64	3.28 ± 0.55	-1.082	-0.163, 0.047	0.280
Avoidance	1.85 ± 0.50	2.06 ± 0.52	-4.521	-0.294, -0.116	< 0.001**
Positive attitude	2.96 ± 0.62	2.70 ± 0.57	4.825	0.154, 0.365	< 0.001**
Overall	2.51 ± 0.37	2.56 ± 0.34	-1.680	-0.116, 0.009	0.094

Data was analyzed using the independent sample t-test.

*Statistical significant level p-value < 0.05

** p-value < 0.001.

### Association between coping strategies and perceived stress symptoms

Multiple logistic regressions were conducted to identify the domains of coping strategies that were used and associated with perceived stress symptoms. **Table 5** depicts the results of adjusted domains, those ho perceived stress symptoms used social support 1.5 times more than those who perceived normal stress (AOR 1.54, 95% CI 1.070–2.236, p-value = 0.02). The avoidance strategy was used 2.03 times in the perceived stress symptoms group, compared to the normal stress group (AOR 2.03, 95% CI 1.406–2.934, p-value < 0.001). Interestingly, the participants who perceived stress symptoms applied a positive attitude strategy less than those who perceived normal stress, 57.5% (AOR 0.42, 95%CI 0.307–0.590, p-value < 0.001).

**Table 5:** Coping strategies associated with perceived stress symptoms.

Domains of coping strategies	B	Exp(B) Adjusted OR	95% CI	p-value
Social support	0.436	1.547	1.070–2.236	0.020*
Avoidance	0.708	2.031	1.406–2.934	< 0.001**
Positive attitude	-0.855	0.425	0.307–0.590	< 0.001**

Perceived Stress Scale (1 = < 21 score (normal), 2 = 21–40 score (perceived stress symptom)).

*Statistical significant level p-value < 0.05

** p-value < 0.001.

## Discussion

This present study measures the prevalence of perceived stress and use of coping strategies among HCWs in a medical school hospital during the third wave of the COVID-19 outbreak, Bangkok metropolitan, Thailand. The result demonstrated that the prevalence of perceived stress symptoms was nearly half (41.97%), which was more than our expected percentage of prevalence in HCWs. Several reasons support this result. Firstly, HCWs have been working up till the third wave and handling patients for a long time during the outbreaks. Secondly, maintaining the new normal in working and living areas, and change in the time of work leads to mental problems. Thirdly, no consensus and assurance of the vaccine’s effectiveness was afforded to them while they waited to receive a COVID vaccine [16]. These possibly caused distress for HCWs because it physically impacted with the burden of care for the continual cases. Moreover, the mental health was severely affected by such uncontrollable events. Although data collection was performed during the third wave, which seemed like a stable situation, this finding confirmed that the long-term effect on mental health still remained. This be in line with a study that mentioned that negative psychological effects of the COVID-19 pandemic had also been reported as post-traumatic stress symptoms [20]. We suggested from this finding that further study should address the assessment of the stress of HCWs after the end of the pandemic.

### Comparison of coping strategies (Brief-COPE score) among HCWs during the COVID-19 pandemic

This study hypothesized the presence of different coping strategies among HCWs based on various socio-demographic characteristics, work factors, and perceived stress. As a result of the comparison of coping strategies based on socio-demographic and work factors. This study indicated that marital status, having children, and having more than eight days off were significant factors for applying coping strategies during an uncontrollable outbreak. HCWs who had partners reported a higher coping score than others. We explain this in the sense that they can deal with stress by seeing their partner as a safe zone; as a means of social and emotional support. This is consistent with a previous study that reported single nurses as having higher stress levels [21]. Besides, support from family, friends, supervisors, and colleagues is said to help reduce mental health disorders [22, 23]. Having children was not only indicated as a predictor of stress during the pandemic but also as a protective factor for stress. We believe this is so because children are among the population vulnerable to infection. In this current study, HCWs who have children reported higher Brief-COPE scores than those who did not have children. A similar finding indicated that the source of worry and stress was about transmitting the disease to their family members including children [24, 25]. On the other hand, the children can be a supporter for relieving stress. This is consistent with a previous study that found that nurses without children reported to have been more stressed [21].

HCWs with different days off reported different coping scores; those with more than normal number of days off (≥ 8 days) reported higher Brief-COPE scores than others. This result probably implies that they have time to relax and learn to live with risk situations. HCWs engaged in activities such as watching TV, spending time with couples, reading, or playing with their children. This was confirmed by the higher score in some of the descriptive items. Some HCWs opted to work from home or limited their number of working days, which granted them more off days. Lockdown was an attempt to limit the transmission of the disease and its transportation, which lead people to live with boring routines [23]. Therefore, HCWs need to manage their emotion and lifestyle under such restricted conditions. These were considered the reasons for using coping strategies. We agreed with a previous study that stated that coping styles were used in the management of the stress of social isolation [26].

### Comparison difference of the use of coping strategies among HCWs during the COVID-19 pandemic

Besides, this study examines that the higher score of Brief-COPE not only means higher stress, but also reflects on the method used to release the associated distress or manage the emotion in poor or risk situations [27]. Overall, from the Brief-Cope score, most HCWs applied the domain of problem-solving and positive attitude. These approach strategies demonstrated that the HCWs could manage their feeling by themselves.

Social support made a significant difference to those who perceived stress symptoms, as opposed to those who perceived normal stress. This has been consistent with prior studies that indicate that the higher the social support needed, the higher the predicted levels of distress [4]. Besides, seeking social support was considered a coping strategy that was used lesser by the participants in the normal mental health group, compared to those in the severe mental health problems group [7]. This probably implies that individuals gain more social support from friends, family, and co-workers when they are faced with problems. We agreed with the statement of a previous study that social support obtained in the form of emotional assistance will become the resource for individuals and facilitate them to deal with stressors and reduce the distress levels [28]. During a pandemic, large-scale social restriction and self-isolation can limit the availability and acceptance of social support although the aim is to stop the spread of the disease [27]. Several studies have emphasized the role of social support in protecting the mental health of various populations, including medical students [28]. Thus, people often feel fear and anxiety, not only with regard to the disease but also the uncertainty of its duration, social restriction, and financial problems that arise consequently.

Avoidance strategy was employed very differently by those who perceived stress symptoms, as opposed to those who perceived normal stress. Based on the fact-finding, negative methods were used fewer times by the Thai HCWs despite having worked at the center of a pandemic. For example, there was a low score of the use of alcohol or other drugs to elevate the mood, make themselves feel good, and get through the burdensome situation. This corroborated with a study on the coping strategies employed by the frontline nursing staff in Alabama during the outbreak where fewer nurses were found to adopt drinking alcohol as a coping strategy [21]. Our evidence agreed with the finding from a previous study that mentioned that substance use was the lowest means reported for coping with stress [29]. Further, these findings support a study from Japan [7] that reported that avoidance strategy was used fewer times by participants in the normal mental health group, compared to those in the severe mental health problems group. We can explain that HCWs preferred to utilize this strategy in a positive dimension. It is not only physical escape, but also mental escape, with the withdrawal of effort, denial, avoidance, and other actions that remove a person from contact with distressing interactions [30].

Positive attitude was employed very differently by those who those perceived stress symptoms, as opposed to those who perceived normal stress. A positive strategy as an emotional approach can change one’s perspectives to one’s own problems. Our findings indicated that HCWs learned to live with, accept the reality of the situation, and look for something good in what was happening. This is consistent with a study on the coping strategies used by the healthcare professionals in the hospitals in Khartoum state [31]. There were some hidden reasons including the fact that in Thailand, the third wave of pandemic surged in gradual trend. HCWs are accustomed to the care of patients or how to manage the procedure. They can work and face the outbreak with continued use of problem-solving and positive thinking strategies. Negative avoidance as a coping strategy, i.e., giving up on coping with the COVID pandemic and self-blaming, were reported at a low level. We can explain that the HCWs used positive avoidances instead such as doing something to stop thinking about the COVID situation or engaging in relaxing activities to take their minds off. This was a proper coping technique. This was similar to a prior study that found that positive coping style was used more than the negative style [25].

Additionally, problem-solving strategy brought no significant difference among those who perceived stress symptoms, as opposed to those who perceived normal stress. The most common technique HCWs employ to cope and take an action to better the situation better is do something that heals their stress. Similarly, trying to face and deal with stressful situations was recommended as a good way to manage the problem [4, 21, 32].

### Association between coping strategies and perceived stress symptoms

This study revealed different scores of the Brief-COPE scores for each coping strategy. The coping strategies that were applied to the group with perceived stress symptoms were adjusted in a model. Social support and avoidance strategies were more frequently used by the participants with perceived stress symptoms.

Regarding social support factor, these are social resources that the HCWs can access. This finding indicated that seeking someone for support and to share feelings is necessary for HCWs who work in a pandemic. This is in parallel with a study of strategies utilized by Japanese HCWs which reported that those who had poor mental health were more likely to adopt the seeking of social support as a coping strategy [7]. Furthermore, this corroborates with a prior study that stated that the COVID-19 situation could cause more stress for the HCWs, and, in turn, lead them to seek more social support as a coping strategy [33]. Similarly, even the study in general population reported that social support was significantly associated with PTSD symptoms [34]. However, this present study contrasted with another study that found that support from family and friends did not necessarily predict higher psychological distress among student cases [28].

Avoidance factor, is one of the families of emotion-focused coping [27]. The present finding presumed that HCWs were likely to face stress. This corroborates with a prior study that mentioned that avoidance strategy was used by those who experienced more stress [3]. Descriptive items demonstrated that HCWs prefer to use the positive to negative avoidance technique. Therefore, learning to avoid some events that induce stress by doing various other activities is an instance of positive avoidance (watching TV, reading, sleeping, or exercising). It was stated that this could be applied to physically leave stressful events, and thus have mental escape from having contact with the distressing interaction [30]. This is in line with a previous study that found that avoiding media news about the COVID-19 such as infection and mortality rates was a possible coping style for a pandemic situation [21]. However, there was a possible disadvantage of emotional management if people continued to keep their distance or avoid situations for a period of time [35]. We recommended that maintaining a balance in the avoidance was necessary. Avoiding should be used for peace mind and to refresh one’s perspective of facing the situation.

Regarding attitude factor, HCWs who perceived normal stress reported a positive attitude score at a higher level. This implies that the perspective or attitude about the situations relates to the level of perceived stress. This corroborates with a previous study that suggested that those who employ a positive attitude as a coping strategy may be less likely to experience psychological distress during the present outbreak [3]. We recommended that a positive attitude should be raised as a program or tactic to encourage HCWs to gain a more positive attitude during the COVID-19 pandemic. The study being mentioned in this section was carried out in medical school hospitals on a large sample size of HCWs who were working in the main area of the pandemic in metropolitan, Thailand. The data was collected during the third wave of the outbreak. Our results demonstrated perceived stress and coping strategies at different times of the pandemic. This is the early study in the metropolitan area of Thailand.

### Limitations and future research

There are several limitations to this study, which is a cross-sectional study conducted through online survey and self-response in under a short time. Although we could gain a large sample size of participants, we did not use a random sampling technique. The result cannot be generalized to describe the stress and coping strategies of HCWs in the provincial areas and private sectors. This study failed to show the different Brief-COPE scores between the group with normal stress and with perceived stress symptoms This study suggested that future study should consider adding more factors such as job satisfaction during the pandemic and vaccination, as well as include other mental distress symptoms. The qualitative study should be addressed to learn more about coping styles of HCWs in metropolitan. To expand this knowledge, the research should be performed on HCWs in private healthcare sectors and rural areas with a high ranking of cases.

## Conclusion

The study illustrated the prevalence of perceived stress symptoms in HCWs in a medical school hospital. Despite the third wave, the findings indicated that the impact of the COVID-19 pandemic on mental distress still remains. Thai HCWs applied coping strategies depending on their condition. In total, problem-solving was used as a dominant strategy. This indicated that HCWs were prompt at facing and learning to live with an unpredictable pandemic. They have sources of power to deal with the cases and situations. Under the Brief-COPE score, the use of coping strategies denoted different scores based on factors such as marital status, having children, and days off. HCWs, who reported perceived stress symptoms used social support and avoidance strategies more than the group that perceived normal stress. Definitely, the approach of positive attitude was used lesser by the group that perceived stress symptoms. The study suggested that each coping strategy is a way to deal with pandemic situations. HCWs should consider merging each of the coping strategies to balance the work and lifestyle under any stressful condition.

## Supporting information

S1 File The full English language version of the questionnaire. The full English language version of the questionnaire contained all the details of the original Thai version of the questionnaire.
(DOCX)

## Abbreviations

CDC: Centers for Disease Control and Prevention
COVID-19: Coronavirus disease
HCWs: Healthcare workers
PSS-10: Perceived Stress Scale
Brief-COPE: Coping strategies
CI: Confidence Interval
