# Supplementary material for: Prevalence of perceived stress and coping strategies among healthcare workers during the COVID-19 outbreak at Bangkok metropolitan, Thailand
Source: PLoS One. 2022 Jul 8;17(7):e0270924. doi: 10.1371/journal.pone.0270924 (PMC9269378; doi:10.1371/journal.pone.0270924)
Supplement: S1 File — The full English language version of the questionnaire contained all the details of the original Thai version of the questionnaire. (DOCX) [file pone.0270924.s001.docx]

**Additional file 1**

The full English language version of the questionnaire. The full English language version of the questionnaire

**Questionnaire (English version)**

**Consent from**

**( ) Allow ( ) Not allow**

**ID ………………**

**The questionnaire is divided into four sections.**

**Section 1.**

1. Gender

( ) Male ( ) Female

1. Age (Years)

( ) ≤ 35 ( ) > 35

1. Education

( ) < bachelor ( ) ≥ bachelor

1. Occupational (Jobs)

( ) Medical staff ( ) Support staff

1. Marital status*

( ) Sigle ( ) Married ( ) Separated

1. Income (Baht)

( ) ≤ 25,000 ( ) > 25,000

1. Having Children

( ) No ( ) Yes

1. Underlying Diseases

( ) No ( ) Yes

1. Family diseases

( ) No ( ) Yes

1. Residence*

( ) Home ( ) Condominium ( ) Hospital

1. Work experience (Years)

( ) ≤ 10 ( ) > 10

1. Work Hours (Days/Month)

( ) ≤8 hours ( ) > 8 Hours

1. Days off (per month)

( ) < 8 ( ) ≥ 8

1. Sleep (hours per day)

( ) ≤ 6 ( ) > 6

1. Number of colleagues

( ) ≤ 3 persons ( ) > 3 persons

1. COVID-19 experienced

( ) Never at risk ( ) Not sure ( ) Experienced risk (ever screening test) ( ) Have been infected

**Section 2.**

Perceived Stress Scale (PSS-10) items

| **Questions PSS-10 items** | | **Never** | **Almost never** | **Sometimes** | **Fairy often** | **Very often** |
| --- | --- | --- | --- | --- | --- | --- |
| 1 | In the last month, how often have you been upset because of something that happened unexpectedly during COVID-19 |  |  |  |  |  |
| 2 | In the last month, how often have you felt that you were unable to control the important things in your life during COVID-19 |  |  |  |  |  |
| 3 | In the last month, how often have you felt nervous and “stressed” during COVID-19 |  |  |  |  |  |
| 4 | In the last month, how often have you felt confident about your ability to handle your personal problems during COVID-19 |  |  |  |  |  |
| 5 | In the last month, how often have you felt that things were going your way during COVID-19 |  |  |  |  |  |
| 6 | In the last month, how often have you found that you could not cope with all the things that you had to do during COVID-19 |  |  |  |  |  |
| 7 | In the last month, how often have you been able to control irritations in your life during COVID-19 |  |  |  |  |  |
| 8 | In the last month, how often have you felt that you were on top of things during COVID-19 |  |  |  |  |  |
| 9 | In the last month, how often have you been angered because of things that were outside of your control during COVID-19 |  |  |  |  |  |
| 10 | In the last month, how often have you felt difficulties were piling up so high that you could not overcome them during COVID-19 |  |  |  |  |  |

**Section 3.**

Coping Strategies (Brief-Cope Score)

| **Questions Brief-Cope Score items** | | I haven't been  doing this at all | A little bit | A medium amount | I’ve been doing  this a lot |
| --- | --- | --- | --- | --- | --- |
| **Social Support** | |  |  |  |  |
| 1. | 1.I've been getting comfort and understanding from someone during COVID-19 |  |  |  |  |
| 2. | 2. I’ve been getting help and advice from other people during COVID-19 |  |  |  |  |
| 3. | 3.I've been saying things to let my unpleasant feelings escape during COVID-19 |  |  |  |  |
| 4. | 4.I've been getting emotional support from others during COVID-19 |  |  |  |  |
| 5. | 5.I’ve been trying to get advice or help from other people about what to do during COVID-19 |  |  |  |  |
| 6. | 6.I've been expressing my negative feelings during COVID-19 |  |  |  |  |
| 7. | 7.I've been praying or meditating during COVID-19 |  |  |  |  |
| 8. | 8.I've been trying to find comfort in my religion or spiritual beliefs during COVID-19 |  |  |  |  |
| **Problem Solving** | |  |  |  |  |
| 9. | 9.I've been taking action to try to make the situation better during COVID-19 |  |  |  |  |
| 10. | 10.I've been concentrating my efforts on doing something about the situation I'm in during COVID-19 |  |  |  |  |
| 11. | 11.I've been trying to come up with a strategy about what to do during COVID-19 |  |  |  |  |
| 12. | 12.I've been thinking hard about what steps to take during COVID-19 |  |  |  |  |
| **Avoidance** | |  |  |  |  |
| 13. | 13.I've been using alcohol or other drugs to help me get through it during COVID-19 |  |  |  |  |
| 14. | 14.I've been using alcohol or other drugs to make myself feel better during COVID-19 |  |  |  |  |
| 15. | 15.I’ve been criticizing myself during COVID-19 |  |  |  |  |
| 16. | 16.I’ve been blaming myself for things that happened during COVID-19 |  |  |  |  |
| 17. | 17.I've been refusing to believe that it has happened during COVID-19 |  |  |  |  |
| 18. | 18.I've been saying to myself "this isn't real" during COVID-19 |  |  |  |  |
| 19. | 19.I've been doing something to think about it less, such as going to movies, watching TV, reading, daydreaming, sleeping, or shopping during COVID-19 |  |  |  |  |
| 20. | I've been giving up the attempt to cope during COVID-19 |  |  |  |  |
| 21. | I've been turning to work or other activities to take my mind off things during COVID-19 |  |  |  |  |
| 22. | I've been giving up trying to deal with it during COVID-19 |  |  |  |  |
| **Positive Attitude** | |  |  |  |  |
| 23 | 23.I've been making jokes about it during COVID-19 |  |  |  |  |
| 24 | 24.I've been making fun of the situation during COVID-19 |  |  |  |  |
| 25 | 25.I've been learning to live with it during COVID-19 |  |  |  |  |
| 26 | 26.I've been accepting the reality of the fact that it has happened during COVID-19 |  |  |  |  |
| 27 | 27.I've been trying to see it in a different light, to make it seem more positive during COVID-19 |  |  |  |  |
| 28 | 28. I've been looking for something good in what is happening during COVID-19 |  |  |  |  |
